# Mechanical ventilation enhances *Acinetobacter baumannii*-induced lung injury through JNK pathways

**DOI:** 10.1186/s12931-021-01739-3

**Published:** 2021-05-22

**Authors:** Tzyy-Bin Tsay, Wan-Hsuan Chang, Ching-Mei Hsu, Lee-Wei Chen

**Affiliations:** 1 Department of Surgery, Kaohsiung Armed Forces General Hospital Zuoying Branch, Kaohsiung, Taiwan; 2 Department of Biological Sciences, National Sun Yat-Sen University, Kaohsiung, Taiwan; 3 Department of Surgery, Kaohsiung Veterans General Hospital, 386, Ta-Chung 1st Road, Kaohsiung, Taiwan; 4 Institute of Emergency and Critical Care Medicine, National Yang Ming Chiao Tung University, Taipei, Taiwan

**Keywords:** BALF, VCAM, IL-6, Nitric oxide, Neutrophil, Alveolar macrophages

## Abstract

**Background:**

Patients in intensive care units (ICUs) often received broad-spectrum antibiotic treatment and *Acinetobacter baumannii (A.b.)* and *Pseudomonas aeruginosa (P.a.)* were the most common pathogens causing ventilator-associated pneumonia (VAP). This study aimed to examine the effects and mechanism of mechanical ventilation (MV) on *A.b.*-induced lung injury and the involvement of alveolar macrophages (AMs).

**Methods:**

C57BL/6 wild-type (WT) and c-Jun N-terminal kinase knockout (JNK1^−/−^) mice received MV for 3 h at 2 days after nasal instillation of *A.b.*, *P.a.* (1 × 10^6^ colony-forming unit, CFU), or normal saline.

**Results:**

Intranasal instillation of 10^6^ CFU *A.b.* in C57BL/6 mice induced a significant increase in total cells and protein levels in the bronchoalveolar lavage fluid (BALF) and neutrophil infiltration in the lungs. MV after *A.b.* instillation increases neutrophil infiltration, interleukin (IL)-6 and vascular cell adhesion molecule (VCAM) mRNA expression in the lungs and total cells, IL-6 levels, and nitrite levels in the BALF. The killing activity of AMs against *A.b.* was lower than against *P.a*. The diminished killing activity was parallel with decreased tumor necrosis factor-α production by AMs compared with *A.b*. Inducible nitric oxide synthase inhibitor, S-methylisothiourea, decreased the total cell number in BALF on mice receiving *A.b.* instillation and ventilation. Moreover, MV decreased the *A.b.* and *P.a.* killing activity of AMs. MV after *A.b.* instillation induced less total cells in the BALF and nitrite production in the serum of JNK1^−/−^ mice than those of WT mice.

**Conclusion:**

*A.b.* is potent in inducing neutrophil infiltration in the lungs and total protein in the BALF. MV enhances *A.b.*-induced lung injury through an increase in the expression of VCAM and IL-6 levels in the BALF and a decrease in the bacteria-killing activity of AMs. A lower inflammation level in JNK1^−/−^ mice indicates that *A.b.*-induced VAP causes lung injury through JNK signaling pathway in the lungs.

**Supplementary Information:**

The online version contains supplementary material available at 10.1186/s12931-021-01739-3.

## Background

*Acinetobacter baumannii* (*A.b.*), a non-fermentative, Gram-negative bacillus, is an opportunistic pathogen that can be easily spread [1]. Patients hospitalized in intensive care units (ICUs) often received broad spectrum antibiotic treatment which may lead to frequent isolation of *A.b.* strain from patients [2, 3]. In 2017, *A.b.* was included in the World Health Organization priority list of antibiotic-resistant bacteria to support research and development of effective drugs [4]. Qiu et al. found that nicotinamide adenine dinucleotide phosphate hydrogen (NADPH) oxidase appeared to play a crucial role in neutrophil-mediated host defense against *A.b.* and to prevent development of severe disease [5]. Early recruitment of neutrophils into the lungs is critical for initiating an efficient host defense against *A.b.* infection [6]. Macrophages may be involved in early host defense against *A.b.* infection through a more efficient phagocytosis and killing of *A.b.*, thus limiting initial pathogen replication and secretion of pro-inflammatory cytokines/chemokines for the rapid recruitment of other innate immune cells such as neutrophils [7]. The management of VAP caused by drug-resistant *A.b.* is still a challenge.

Mechanical ventilation (MV) is a lifesaving intervention for patients with respiratory distress. It can maintain normal oxygen supply and improve survival. Typically, MV is administered to at least 40% of the patients in ICUs in the United States [8]. However, prolonged ventilation or excessive tidal volume can cause serious lung injury in patients, called ventilator-associated lung injury [9] that intensifies lung injury or inflammation and thus increases patient mortality rate [11]. Experimental models demonstrated increased vascular permeability, higher cell count and protein concentration in the bronchoalveolar lavage fluid (BALF) and increased infiltration of inflammatory cells into lung issues in patients with ventilator-induced lung injury patients. Other investigations have also revealed that cytokine/chemokine production, protein leakage, phagocytic response, pulmonary edema and atelectasis could be indications of lung injury [12, 13]. Ventilator-associated pneumonia (VAP) is the most frequent infection in ICUs. *A.b.* and *Pseudomonas aeruginosa* (*P.a.*), which particularly resist carbapenem antibiotics, were reported to be major agents and may cause important therapeutic challenges [14]. Our previous data suggested that the pathogenetic mechanism of VAP caused by *P.a.* involves tumor necrosis factor (TNF)-α production through the activation of NF-κB in alveolar macrophages (AMs) and JNK signaling pathway in lung tissues [15]. van Faassen et al. found that the increased susceptibility to *A.b.* of neutropenic mice was associated with a delay in the mRNA expression and production of early pro-inflammatory cytokines in the lungs. These results imply that neutrophils play a critical role in host resistance to respiratory *A.b.* infection [16]. However, the effects and mechanism of MV on *A.b.*-induced lung injury has not been characterized.

Airway epithelial cells are the frontline defenders of the lungs against invading microbes by providing a physical barrier and antimicrobial activity [17]. Airway epithelial cells increase the production of mediators such as cytokines, chemokines and antimicrobial peptides in response to such exposure [18]. In response to pathogens, endothelial cells promote inflammation by expressing different combinations of adhesion molecules for leukocytes such as E-selectin, intercellular adhesion molecule-1 and vascular cell adhesion molecule-1 (VCAM-1) in distinct temporal, spatial and anatomical patterns [19]. In this study, nasal instillation of *A.b.* before MV in mice was used as a model to study the mechanism of *A.b.* VAP-induced lung injury.

The objectives of this study were as follows: [1] to determine the mechanism of *A.b.*-induced lung injury, [2] to examine the effects of MV on *A.b.*-induced lung injury and the involvement of AMs, and [3] to examine the involvement of JNK signaling pathways in *A.b.* VAP-induced lung injury. Our results suggest that MV enhances *A.b.*-induced lung injury by decreasing the killing activity of AMs and increasing expression of VCAM in the lungs and inflammatory mediators in the BALF.

## Methods

### Animals

Male C57BL/6 (wild-type, WT) mice weighing from 18 to 25 g were purchased from National Laboratory Breeding and Research Center (NLBRC, Taipei, Taiwan). JNK1^−/−^ (c-Jun N-terminal kinases knockout) mice generated from the same background were transferred from Dr. Karin’s laboratory (University of California, San Diego, CA, USA). All animals were housed in a temperature controlled room for at least one week before the experiments. All animal procedures were in compliance with regulations on animals used for experimental and other scientific purposes approved by the National Sun Yat-Sen University Animal Experiments Committee.

### Experimental design

In experiment 1, the animal model of VAP-induced by *A.b.* or *P.a*. was established. WT mice were anesthetized, instilled with live *A.b.* or *P.a.* intranasally, and received MV for 3 h at 2 days after bacterial instillation. Lung tissues were harvested and assayed for the expression of pro-inflammatory cytokines/chemokines, and BALFs were collected for cell counting and nitrite, protein and cytokine assay.

In experiment 2, WT mice receiving *A.b.* instillation were given inducible nitric oxide synthase (iNOS) specific inhibitor, S-methylisothiourea (SMT) [20], injection (7.5 mg/kg, i. p.) at 1 or 2 h before ventilation to evaluate the effect of iNOS inhibition on *A.b.* VAP-induced lung injury. Lung tissues and BALFs were harvested and assayed as described in experiment 1.

JNK1 is the major mediator of iNOS induction [21]. In experiment 3, JNK1^−/−^ mice were used to study the role of JNK signaling pathways in *A.b.*-induced VAP. Lung tissues were harvested and assayed as described in experiment 1.

### Nasal instillation of mice with *A.b.* or *P.a.*

Mice were anesthetized with avertin (15 mg/kg) and slowly instilled with 10 μl of saline containing 1 × 10^6^ colony-forming units (CFU) of live *A.b.* (a gift from Dr. Te-Li Chen at Taipei Veterans General Hospital in Taiwan), or *P.a.* (ATCC 9027), or sterile saline (as control) into the lungs via the nostrils. Two days later, mice were sacrificed or received MV for 3 h, and the lungs and BALF were collected for assay.

### Mechanical ventilation treatment

At 2 days after instillation, the mice were sacrificed or received mechanical ventilation for 3 h. Mice were anesthetized with Avertin (15 mg/kg, Sigma), and the neck was cut at 1 cm below the mouth. The muscles were separated and the trachea was opened and cannulated with a 0.5 cm 21G needle connected to a mechanical ventilator (SAR-830/P, CWE Inc., Ardmore, PA, USA) with an analog pressure output signal for 3 h. Mice were administered avertin every 20 min during the period of ventilation. The ventilation was with high stretch (tidal volume, Vt = 30 ml/kg) and without positive end expiratory pressure (PEEP).

### Tissue preparation

Mice were sacrificed and the lungs and heart were harvested. Saline (5 ml) was injected into the right ventricle by syringe to clear the blood in pulmonary vasculature. The lung tissue was blotted dry to remove the blood on the surface and immediately stored at -80 °C for later use.

### Bronchoalveolar lavage fluid (BALF) collection

Lungs were lavaged twice with 0.5 ml of sterile saline through a tracheal cannula made by a 21G needle and the BALF was placed into an eppendorf. The number of cells in BALF was counted by using the hemocytometer. The collected BALF was centrifuged at 350 × g, room temperature for 5 min, and the pellet (cells) was used for ex vivo alveolar macrophage stimulation assay or RT-PCR assay. The supernatant was analyzed by Griess assay to quantify nitrite production immediately or frozen at -80 °C for Enzyme-Linked immunosorbent assay (ELISA) for cytokine production and for total protein assay.

### Neutrophil infiltration of the lungs

Lung myeloperoxidase (MPO) activity has been used as a marker of lung neutrophil infiltration [22]. Lung tissues were weighed and homogenized in 50 mM potassium phosphate buffer (pH 6.0) with 0.5% hexadecyltrimethyl-ammonium bromide. Homogenates were centrifuged at 9500×*g*, 4 °C for 10 min. An aliquot (60 μl) of supernatants was added to 939 μl of potassium phosphate buffer with 16.7 mg/ml of O-dianisidine and 0.5% hydrogen peroxide. The rate of change in absorbance at 460 nm was measured over 2 min. One unit of MPO activity is defined as the amount of enzyme that reduces 1 μmole of peroxide per min and the data were expressed as units per gram of lung tissue (Units/g tissue).

### Griess assay for nitrite production

NO is a mediator of inflammation. The levels of NO are determined by assaying the nitrite level with Griess reaction as described [23]. Equal volumes of N-[1-naphthyl]-ethylenediamine (Sigma-Aldrich) and sulfanilic acid (Sigma-Aldrich) are freshly mixed to form the Griess Reagent. The samples (100 μl/well) and a serial dilution of standards (100 mM NaNO_2_) were put into the microplate and added the prepared Griess reagent (40 μl/well). The plate was incubated at room temperature for 20 min in the dark. The absorbance at 550 nm was measured by using an ELISA reader.

### *Assay for *ex vivo* stimulation of alveolar macrophages*

Pellets collected after centrifugation of BALF were suspended with RPMI 1640 (Sigma) in 96-well microtiter plates (200 μl/well) and cultured at 37 °C for 2 h for attachment. Nonattached cells were washed away and the attached cells (AMs) were stimulated with or without live *P. aeruginosa* or *A. baumannii* (10^6^ CFU in 200 μl) at 37 °C for 4 h. After stimulation, supernatants were collected, incubated in 65 °C water bath for 1 h, and centrifuged at 9,500 × g for 15 min to remove bacterial cells. The supernatant samples were used for TNF-α levels by ELISA.

### Ex vivo* bacterial killing activity of alveolar macrophages*

The attached AMs were given 200 μl of bacterial suspension containing live *A. baumannii* or *P. aeruginosa* (10^6^ CFU) and incubated at 37 °C for 30 min. After incubation, 100 μl of supernatant was diluted and plated onto LB agar plates. The plates were examined for bacterial growth after overnight aerobic incubation at 37 °C. Data were expressed as the percentage when compared to the groups without AMs.

### Polymerase chain reaction (PCR) and quantitative real-time PCR

Total RNAs were extracted from lung tissues or cells from BALF by using the Miniprep Purification Kit (GeneMark). cDNAs encoding pro-inflammatory cytokines and chemokines were generated by reverse transcription and amplified by PCR. Glyceraldehyde-3-phosphate dehydrogenase (GAPDH) gene is used as the control and sets of TNF-α, iNOS, IL-1β, IL-6, ICAM, VCAM, CXCR-2, MIP-2 and GADPH primers were designed according to which documented in the GenBank (additional file 1).

For PCR reactions, 0.2 ml tubes were added 3 μl of 10X Gene Taq buffer (GeneMark Inc., Atlanta, GA, USA), 2 μl of 2.5 mM dNTP, 0.5 μl of 25 mM sense and antisense primers and water to make a total volume of 30 μl. Each tube was added 0.05 μl of Gene Taq DNA polymerase (5 U/μl). The amplification was performed in a thermocycler (Bio-Rad) with the following profile: 5 min at 95 °C before the first cycle, 1 min at 95 °C for denaturation, 1 min at 58 °C for annealing, and 1 min 30 s at 72 °C for extension, finally 10 min at 72 °C after the last cycle. The PCR products were separated on a 1.5% agarose gel and stained with ethidium bromide. The approximate sizes of PCR products were obtained by comparing with the markers (100 bp Ladder, New England Biolabs, Beverly, MA, USA).

### Enzyme-linked immunosorbent assay (ELISA)

Lung tissues, BALF and supernatants collected after ex vivo stimulation of AMs were assayed for TNF-α, IL-1β and IL-6 production by using the mouse ELISA kit (eBioscience). Lung tissues were homogenized in lysis buffer (30 mM Tris, pH 7.5, 300 mM NaCl, 2 mM MgCl_2_, 10% Triton X-100, 2 mM CaCl_2_, and 20 μg/ml of protease inhibitor) and centrifuged at 1000×*g*, 4 °C for 15 min. The supernatants were collected and used for assay. The ELISA plates were coated with capture antibodies (100 μl/well) at 4 °C for overnight, then washed several times and blocked with assay buffer (200 μl/well) at room temperature for 1 h. The samples and standards were added to the plates and incubated at 4 °C for overnight, then, the plates were washed several times. Detection antibodies (100 μl/well) were added for 1 h and avidin-HRP (100 μl /well) was added for 30 min at room temperature. Finally, substrate 3,3',5,5'-tetramethylbenzidine was added and incubated at room temperature for 15 min. The reaction was stopped by adding 2 N H_2_SO_4_ (50 μl/well) and the absorbance at 450 nm was measured by using an ELISA reader.

### Histological study

Tissue samples were collected and fixed in 4% formalin for 24 h. The samples were embedded in paraffin, cut into 3–5 μm sections, and stained with hematoxylin and eosin. Infiltration of inflammatory cells and alveolar wall thickening were observed.

### Statistics

All data are analyzed by one-way analysis of variance or T-test analysis of variance (ANOVA), followed by Turkey’s Multiple Comparison Test. All values in the figures and text are expressed as mean ± standard error of the mean. P values of less than 0.05 are considered to be statistically significant.

## Results

### MV enhances *A.b.*-induced neutrophil infiltration in the lungs

**Fig. 1:**
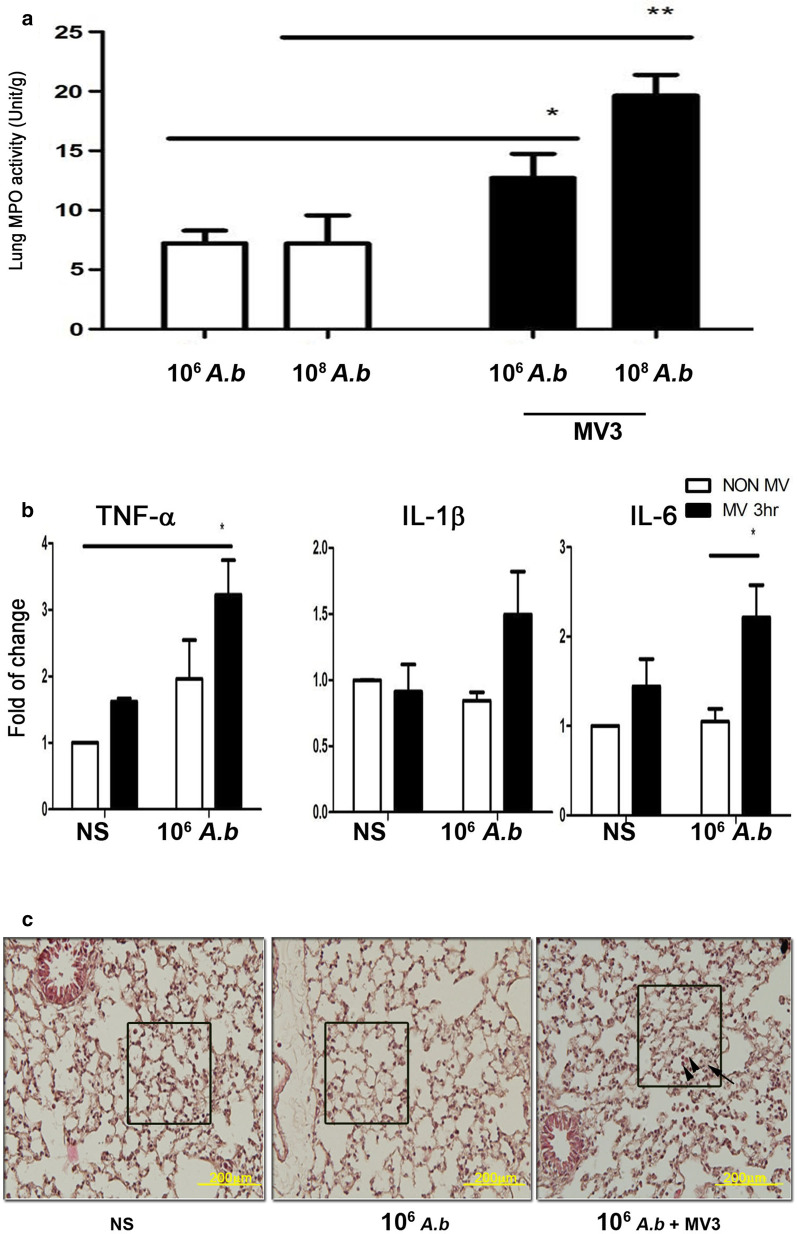
Mechanical ventilation enhanced *Acinetobacter baumannii (A.b.)*-induced MPO activity and IL-6 expression in lung tissues. **a** Mice receiving instillation of 10^8^ CFU of *A.b.* after ventilation demonstrated a significant increase of the MPO activity in the lungs compared with instillation of 10^8^ CFU of *A.b.* **b** Expression of TNF-α, IL-1β and IL-6 mRNA in the lungs of WT mice receiving *P.a.* or *A.b.* instillation and ventilation. Levels of mRNA expression were detected by real-time PCR. WT mice were intranasal instilled with 1 × 10^6^ CFU of live *A.b.* and received mechanical ventilation for 3 h at 2 days after bacterial instillation. **c** Histological examination of the lungs indicated MV after instillations of 10^6^ *A.b.* caused significant neutrophil infiltration (arrowhead) and alveolar swelling (arrow) in the lungs. *MPO* myeloperoxidase, *MV* mechanical ventilation, *BALF* bronchoalveolar lavage fluid, *TNF* tumor necrosis factor, *IL* interleukin, *CFU* colony-forming unit, *PCR* polymerase chain reaction; *WT* wild type, *AL* alveolar lumen, wild type, *NS* normal saline. *P < 0.05, **P < 0.01

To investigate the effects of MV on *A.b.*-induced neutrophil infiltration in the lungs, pulmonary myeloperoxidase (MPO) activity was examined in different groups. MV after *A.b.* instillation of 10^6^ or 10^8^ CFU significantly increased neutrophil infiltration in the lungs by two- (12.72 ± 4.03 vs 7.23 ± 2.11 unit/g) and three-folds (19.64 ± 2.46 vs 7.21 ± 4.09 unit/g) compared with 10^6^ or 10^8^ CFU of *A.b.* instillation (Fig. 1a). These results suggest that MV enhances *A.b.*-induced neutrophil infiltration in the lungs.

### MV enhances *A.b.*-induced interleukin (IL)-6 expression and alveolar wall thickening in the lungs

To examine the effects of MV on *A.b.*- or *P.a.*-induced inflammatory cytokine expression in the lungs, expression of tumor necrosis factor (TNF)-α, IL-1β, and IL-6 mRNA in the lungs were examined in different groups. Either *A.b.* instillation or MV did not significantly induce the expression of TNF-α, IL-1β, and IL-6 in the lungs compared with saline instillation. MV significantly increased the expression of TNF-α mRNA in the lungs of the *A.b.* + MV group by two-folds compared with the saline + MV group (Fig. 1b). IL-6 expression in the lungs of the *A.b.* + MV group increased approximately two-folds compared with that in the 10^6^ *A.b.* group (Fig. 1b). Histological examination of the lungs indicated ventilation with 10^6^ *A.b.* instillation caused infiltration of neutrophils into the lung tissue and alveolar wall thickening compared with *A.b.* instillation. (Fig. 1c). These results suggest that MV enhances *A.b.*-induced IL-6 mRNA expression, neutrophil infiltration, and alveolar wall thickening in the lungs.

### MV enhances bacteria-induced neutrophil infiltration in the lungs

To determine the effects of MV on *A.b.*- or *P.a.*-induced neutrophil infiltration in the lungs, pulmonary MPO activity was examined in mice receiving 10^6^ *A.b.* or 10^6^ *P.a.* instillation. MV significantly enhanced 10^6^ *A.b.*-induced neutrophil infiltration in the lungs by two-folds compared with those received 10^6^ *A.b.* instillation (12.72 ± 4.03 vs 7.23 ± 2.11 unit/g). MV did not significantly enhance 10^6^ *P.a.*-induced neutrophil infiltration in the lungs compared with those received 10^6^ *P.a.* instillation (9.54 ± 2.34 vs 4.7 ± 1.06 unit/g). These results suggest that MV after *A.b.* instillation enhances *A.b.*-induced neutrophil infiltration in the lungs.

### MV enhances *A.b.*-induced total cells in the BALF

**Fig. 2:**
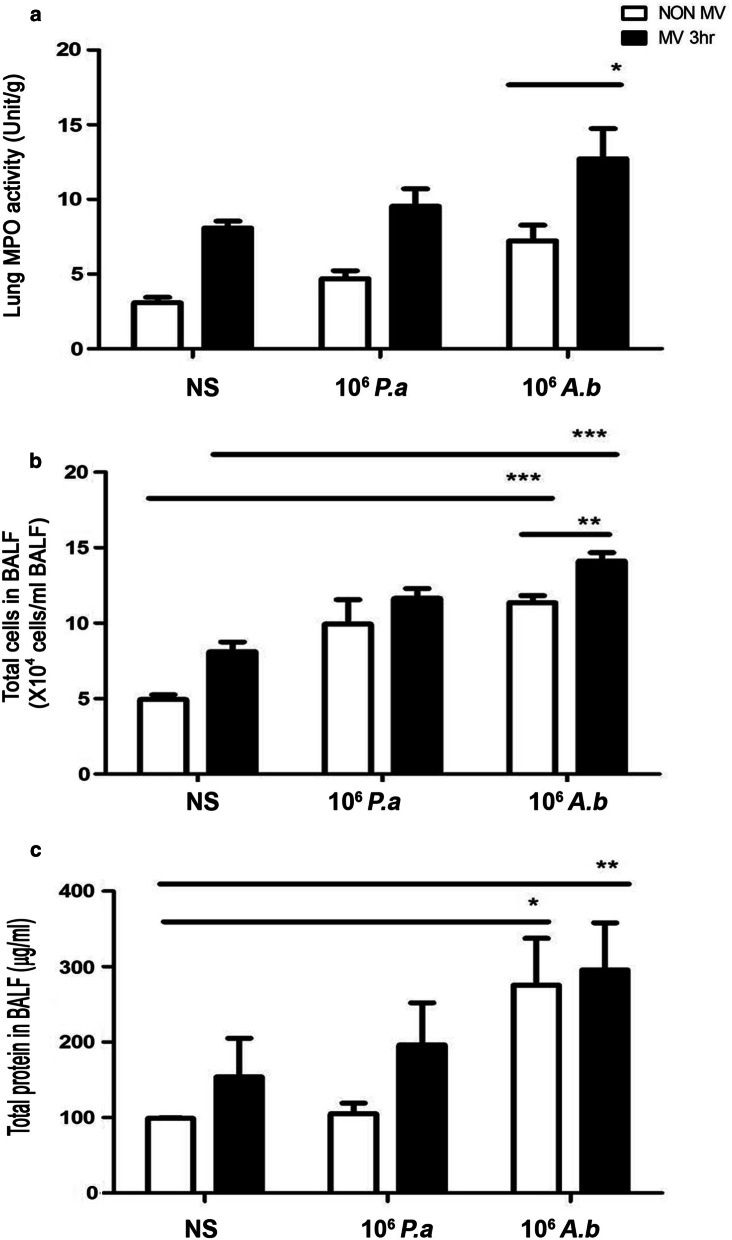
Mechanical ventilation enhanced *Acinetobacter baumannii* instillation-induced total cells and protein in the BALF. **a** The effects of mechanical ventilation on 10^6^ *P. aeruginosa (P.a.) or* 10^6^ *A. baumannii (A.b.)* instillation-induced neutrophil infiltration in the lung. WT mice instilled with live *P.a., A.b.,* or normal saline via nostrils 2 days before receiving ventilation for 3 h and lung tissues were collected and analyzed. MV after instillations of 10^6^ *A.b.* or 10^6^ *P.a.* increased the total cell number **b** and protein concentrations **c** in the BALF of WT mice. n = 5–6/group. *MPO* myeloperoxidase, *BALF* bronchoalveolar lavage fluid, *WT* wild type, *MV* mechanical ventilation, *NS* normal saline. *P < 0.05, **P < 0.01, ***P < 0.001

To investigate the effects of MV on *A.b.*- or *P.a.*-induced total cells in the BALF, the total cells in the BALF were examined in mice receiving 10^6^ *A.b.* or 10^6^ *P.a.* instillation. Intranasal instillation of 10^6^ *A.b.* significantly increased the total cells in the BALF by two-folds compared with normal saline instillation (11.35 ± 1.05 vs 4.95 ± 0.69 × 10^4^ cells/ml BALF) (Fig. 2b). The number of cells in the BALF represents the migration of AMs to defend against the invading microorganisms. MV significantly enhanced 10^6^ *A.b.*-induced total cells in the lungs compared with 10^6^ *A.b.* instillation (14.1 ± 1.23 vs 11.35 ± 1.05 × 10^4^ cells/ml BALF). Instillation of 10^6^ *A.b.* before MV also induced a significant increase in the number of total cells in the lungs compared with saline instillation + MV group (14.1 ± 1.23 vs 8.1 ± 1.46 × 10^4^ cells/ml BALF). These results indicate that intranasal instillation of 10^6^ *A.b.* increases the number of total cells in the BALF and MV after *A.b.* instillation enhanced them.

### MV enhances *A.b.*-induced total protein levels in the BALF

Many experimental studies have used BALF total protein to evaluate lung injury [24]. To explore the effects of MV on *A.b.*- or *P.a.*-induced total protein levels in the BALF, total protein levels in the BALF were examined in mice receiving 10^6^ *A.b.* or 10^6^ *P.a.* instillation. The total protein concentrations in the BALF indicate the production of inflammatory cytokines/chemokines in the airway. Intranasal instillation of 10^6^ *A.b.* significantly increased the total protein levels in the BALF by two-folds compared with normal saline instillation (275.55 ± 123.79 vs 98.99 ± 0.69 μg /ml BALF) (Fig. 2c). These results indicate that intranasal instillation of *A.b.* induces total protein levels in the BALF.

### MV induces VCAM expression in the lungs

**Fig. 3:**
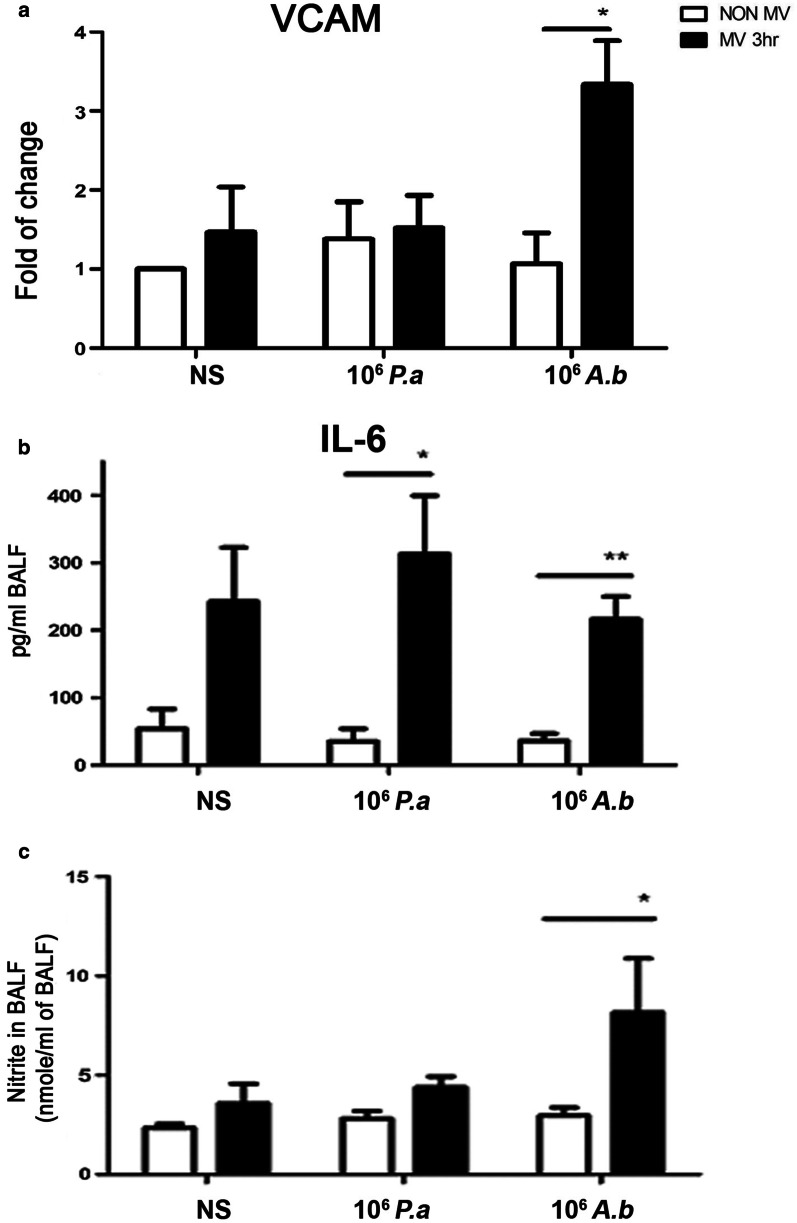
Mechanical ventilation enhanced *Acinetobacter baumannii (A.b.)* instillation-induced VCAM in the lungs and IL-6 levels and nitrite levels in the BALF. **a** Expression of VCAM mRNA in the lungs of WT mice receiving *P. aeruginosa (P.a.)* or *A.b.* instillation and ventilation. **b** IL-6 levels in the BALF of WT mice on MV after 10^6^ CFU *A.b.* or 10^6^ CFU *P.a.* instillation. Concentrations of IL-6 in the BALF were detected by ELISA. **c** Nitrite production in the BALF. 10^6^ CFU *A.b.* instillation induces an increase in the nitrite production in the BALF. *WT* wild type, *MV* mechanical ventilation, *VCAM* vascular cell adhesion molecule, *BALF* bronchoalveolar lavage fluid, *NS* normal saline. *P < 0.05, **P < 0.01

To examine the effects of MV on *A.b.*- or *P.a.*-induced expression of VCAM mRNA in the lungs, pro-inflammatory cytokine/chemokine mRNA in the lungs were examined in different groups. MV, 10^6^ *A.b.,* or 10^6^ *P.a.* instillation did not induce VCAM mRNA expression in the lungs. MV after 10^6^ *A.b.* instillation significantly induced VCAM mRNA expression in the lungs compared with *A.b.* instillation (Fig. 3a). These results indicate that MV enhances *A.b.* instillation-induced VCAM mRNA expression in the lungs, whose lung expression was closely related to MV rather than to *A.b. or* 10^6^ *P.a.* instillation.

### MV induces IL-6 levels in the BALF

To study the effects of MV on *A.b.*- or *P.a.*-induced cytokine levels in the BALF, the concentrations of IL-6 in the BALF were examined in different groups. Instillation of 10^6^ *A.b.* or 10^6^ *P.a.* did not induce IL-6 levels in the BALF compared with normal saline instillation (Fig. 3b). However, MV after 10^6^ *A.b. or* 10^6^ *P.a.* instillation significantly increased IL-6 levels in the BALF compared with 10^6^ *A.b. or* 10^6^ *P.a.* instillation (Fig. 3b). These results indicate that MV after bacterial instillation increases IL-6 levels in the BALF, whose levels were highly related to ventilation rather than to *A.b. or* 10^6^ *P.a.* instillation.

### MV enhances *A.b.*-induced nitrite levels in the BALF

To determine the effects of MV on 10^6^ *A.b.*- or *P.a.*-induced lung inflammation, BALF was collected for examination of nitrite levels. Instillation of 10^6^ *A.b.* or 10^6^ *P.a.* did not induce nitrite levels in the BALF compared with normal saline instillation. However, MV after 10^6^ *A.b.* instillation significantly increased nitrite levels in the BALF compared with 10^6^ *A.b.* instillation (6.64 ± 5.55 vs 2.70 ± 0.79 nmole/ml BALF) (Fig. 3c). MV after 10^6^ *P.a.* instillation did not significantly increase nitrite levels in the BALF compared with 10^6^ *P.a.* instillation (4.5 ± 1.36 vs 2.89 ± 0.98 nmole/ml BALF). These results suggest that MV after 10^6^ *A.b.* instillation significantly increased nitrite levels in the BALF and nitrite is an important mediator in the lung injury caused by MV after *A.b.* instillation.

### MV decreases the activity of AMs

**Fig. 4:**
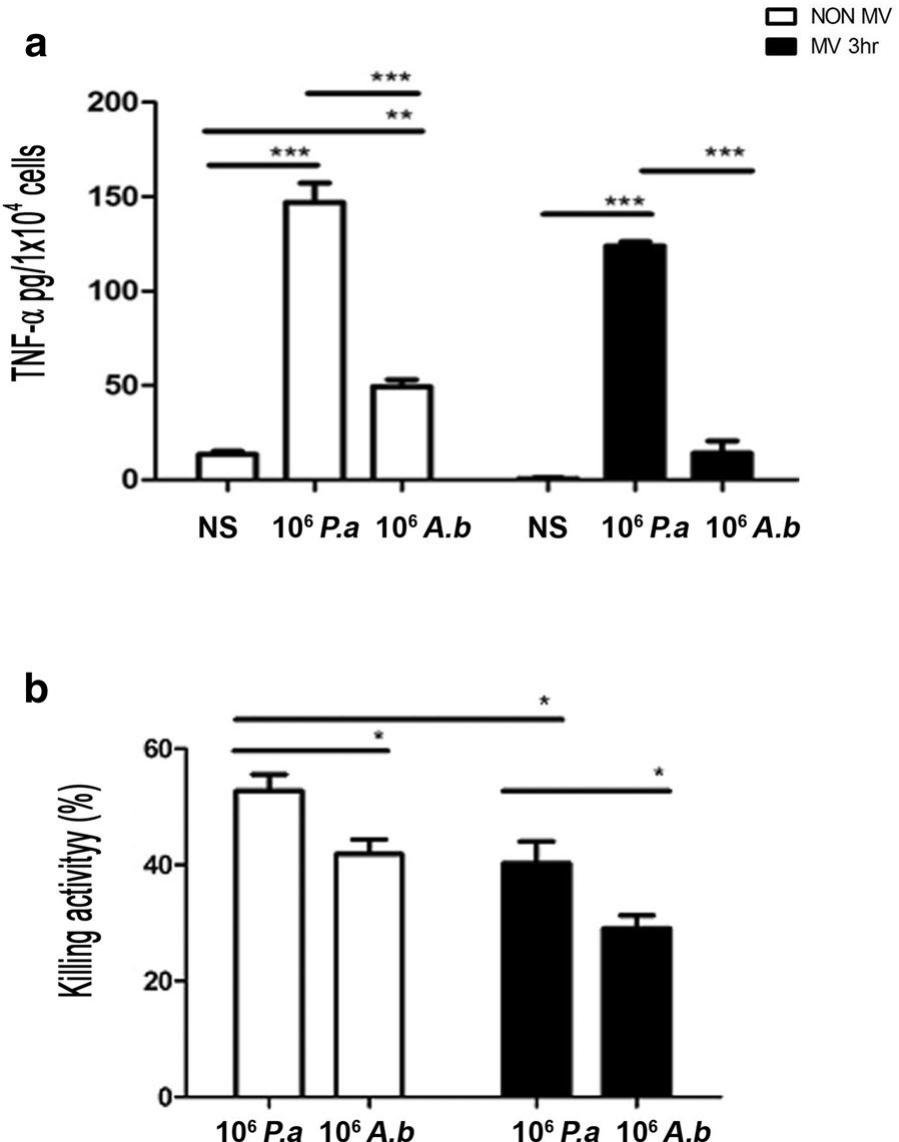
Mechanical ventilation reduces bacteria-killing activity and activity of alveolar macrophages (AMs). **a** The production of TNF-α by AMs was significantly increased after ex vivo stimulation with *A. baumannii (A.b.)* or *P. aeruginosa (P.a.)*. *P.a.* induced three-folds production of TNF-α by AMs compared with that of *A.b.* After ventilation, TNF-α production by AMs were all decreased with or without stimulation by *A.b. or P.a.* AMs isolated from mice were ex vivo stimulated with *A.b. or P.a.* (10^6^ CFU) for 4 h and the supernatants were examined. The concentrations of TNF-α were determined by enzyme-linked immunosorbent assay. n = 5/group. **b** The bacteria-killing ability of AMs isolated after ventilation was markedly decreased compared with the control group. The killing ability against *A.b.* was constantly and significantly lower than against *P.a.* before or after ventilation. *BALF* bronchoalveolar lavage fluid, *MV* mechanical ventilation, *TNF* tumor necrosis factor, *NS* normal saline. *P < 0.05, **P < 0.01, ***P < 0.001. n = 5–6/group

To determine the effects of 10^6^ *A.b.* or 10^6^ *P.a.* with or without MV on the activity of AMs, AMs were harvested and treated with *A.b.* or *P.a*. The production of TNF-α by AMs was significantly increased after ex vivo stimulation with *A.b.* or *P.a.* (Fig. 4a). *P.a.* induced three-folds production of TNF-α by AMs compared with that of *A.b*. After ventilation, TNF-α production by AMs were all decreased with or without stimulation by *A.b.* or *P.a.* Moreover, TNF-α production stimulated by *P.a.* was decreased by 16%, and production stimulated by *A.b.* was decreased by 72% after MV compared with *P.a.* or *A.b*. stimulation. These results indicate that *A.b.* or *P.a.* treatment stimulates the activity of AMs, *P.a.* is more potent in stimulating the activity of AMs than *A.b*., and MV decreases the activity of AMs.

### MV reduces the bacteria-killing ability of AMs

To determine the effects of MV on the bacteria-killing activity of AMs, AMs were harvested with ex vivo incubation of live suspended 10^6^ CFU *A.b.* or *P.a.* Our data showed that the *P.a.*-killing ability of AMs isolated after MV was markedly decreased compared with that in the control group (Fig. 4b). Interestingly, the killing ability of AMs against *A.b.* was constantly and significantly lower than those against *P.a.* These results suggest that MV decreases the bacteria-killing activity of AMs and that AMs have better killing activity against *P.a.* than against *A.b.*

### Inducible nitric oxide synthase (iNOS) inhibitor decreased the total cell number in BALF

**Fig. 5:**
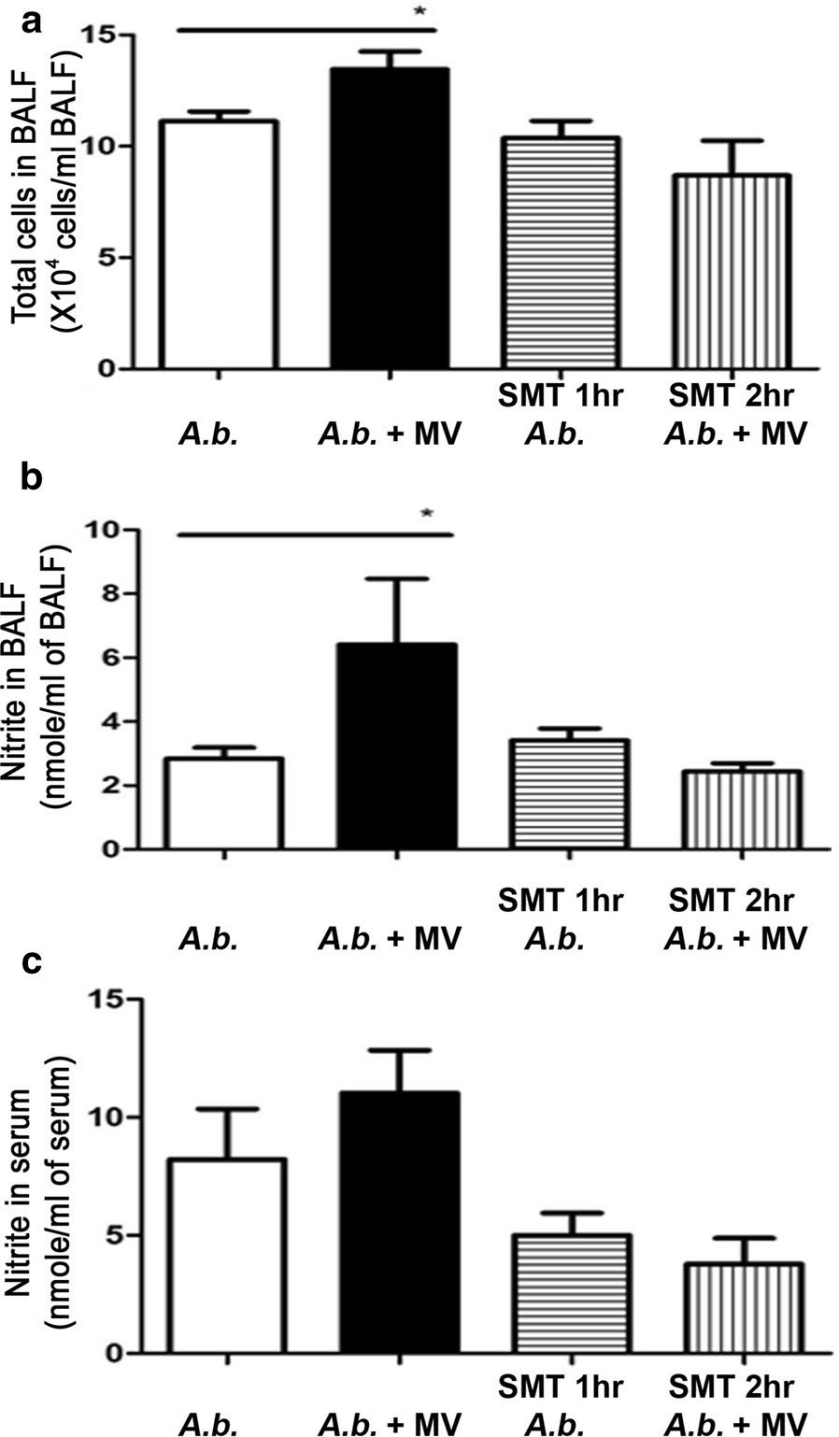
*Acinetobacter baumannii (A.b.)* instillation with S-methylisothiourea (SMT) injection before ventilation decreased the total cell number in BALF of WT mice. **a** SMT is used to investigate the role of iNOS in lung injury induced by *A.b.* instillation after ventilation. SMT treatment significantly decreased the total cell number in BALF, indicating that SMT injection before ventilation could prevent the inflammation in BALF. The production of nitrite in BALF **b** was inhibited by SMT injection at 1 h before ventilation and further decreased 30% by SMT injection at 2 h before ventilation. Also, the level of nitrite in serum **c** as decreased 50% by SMT injection at 1 h before ventilation and 65% by SMT injection at 2 h before ventilation respectively. *BALF* bronchoalveolar lavage fluid, *MV* mechanical ventilation, *NS* normal saline. *P < 0.05. n = 5–6/group

S-methylisothiourea (SMT) is used to investigate the role of iNOS in lung injury induced by *A.b.* instillation after ventilation. SMT injection had no effect on the lung MPO activity after ventilation. SMT treatment significantly decreased the total cell number in BALF (Fig. 5a), indicating the recruitment of cells into airway was decreased. The production of nitrite in BALF was inhibited by SMT injection at 1 h before ventilation and further decreased 30% by SMT injection at 2 h before ventilation (Fig. 5b). Also, the level of nitrite in serum was decreased 50% by SMT injection at 1 h before ventilation and 65% by SMT injection at 2 h before ventilation respectively (Fig. 5c). Altogether, these results suggest that iNOS inhibitor decreased the total cell number in BALF on mice receiving *A.b.* instillation and ventilation.

### MV induces less ***A.b.***-induced total cells in the BALF in JNK1^−/−^ mice than in WT mice

**Fig. 6:**
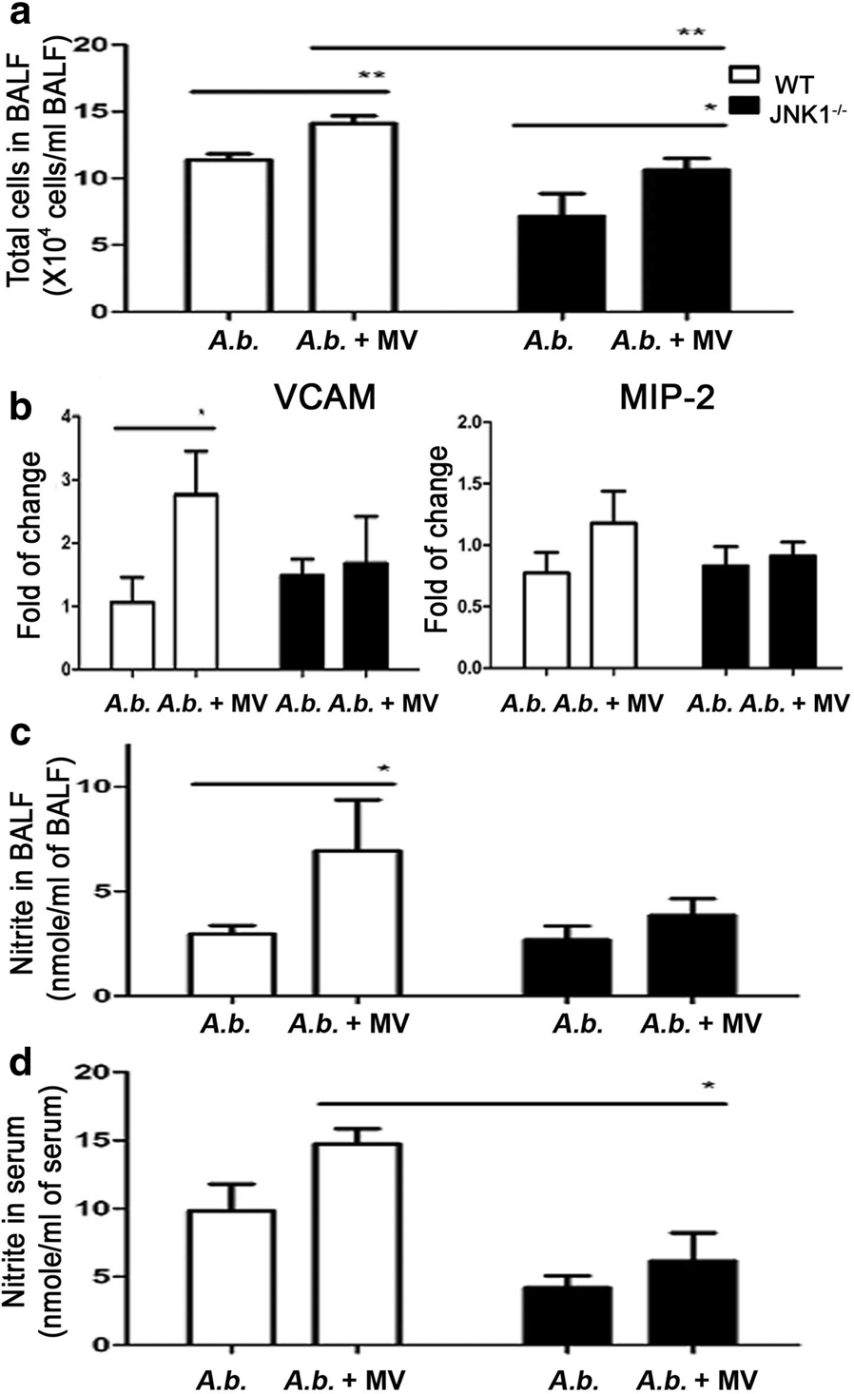
Mechanical ventilation did not increase *Acinetobacter baumannii (A.b.)*-induced total cells and nitrite in the BALF, VCAM and MIP-2 expression in the lungs, and nitrite levels in the serum of JNK1^−/−^ mice than those of WT mice. **a** Total cell counts in the BALF were increased in JNK1^**−/−**^ mice receiving *A.b.* and ventilation. **b** Expression of pro-inflammatory chemokines and adhesion molecule mRNA in the lungs of WT and JNK1^−/−^ mice receiving *A.b.* instillation and ventilation. VCAM expression was lower in the lungs of JNK1^−/−^ mice than that of WT mice after *A.b.* instillation and ventilation. **c**, **d** The nitrite levels in the BALF and serum of WT and JNK1^−/−^ mice receiving *A.b.* instillation and ventilation. Nitrite production in the BALF and serum was decreased in JNK1^−/−^ mice. *VCAM* vascular cell adhesion molecule, *BALF* bronchoalveolar lavage fluid, *NS* normal saline. *P < 0.05, **P < 0.01, ***P < 0.001

To determine the involvement of JNK1 signaling pathways in the effects of MV *A.b.*-induced lung injury, total cells in the BALF in JNK1^−/−^ mice were examined. MV after 10^6^ *A.b.* instillation induced a significant increase in total cells in the BALF in WT and JNK1^−/−^ mice compared with 10^6^ *A.b.* instillation (14.2 ± 1.23 and 10.63 ± 1.74 vs 11.35 ± 1.05 and 7.17 ± 2.93 × 10^4^ cells/ml BALF). MV after 10^6^ *A.b.* instillation induced more total cells in the BALF in WT mice than in JNK1^−/−^ mice (14.2 ± 1.23 vs 10.63 ± 1.74 × 10^4^ cells/ml BALF) (Fig. 6a). These results suggest that the JNK1 signaling pathway is critical to the stimulatory effect of MV on *A.b.*-induced total cells in the BALF.

### MV enhances *A.b.*-induced VCAM expression in WT mice but not in JNK1^−/−^ mice

To determine the involvement of JNK1 signaling pathways in the effects of MV with or without *A.b.* instillation on lung inflammation, VCAM and MIP-2 mRNA expression of the lung tissues in JNK1^−/−^ mice was examined. MV significantly enhanced VCAM mRNA expression in the lungs in WT mice but not in JNK1^−/−^ mice (Fig. 6b). These results suggest that the JNK1 signaling pathway is critical to the stimulatory effect of MV-induced VCAM and MIP-2 mRNA expression in the lungs.

### MV enhances *A.b.*-induced nitrite levels in the BALF in WT mice but not in JNK1^−/−^ mice

To determine the involvement of JNK1 signaling pathways in the stimulatory effects of MV on inflammation in the BALF, nitrite levels in the BALF in JNK1^−/−^ mice were examined. MV after 10^6^ *A.b.* instillation significantly enhanced nitrite levels in the BALF compared with *A.b.* instillation in WT mice (6.64 ± 5.55 vs 2.70 ± 0.79 nmole/ml BALF) but not in JNK1^−/−^ mice (3.97 ± 2.17 vs 2.70 ± 1.84 nmole/ml BALF) (Fig. 6c). MV with 10^6^ *A.b.* instillation induced a significant increase in nitrite levels in the serum of WT mice compared with those of JNK1^−/−^ mice (14.77 ± 1.90 vs 4.20 ± 1.76 nmole/ml BALF) (Fig. 6d). These results suggest that MV enhances *A.b.*-induced nitrite levels in the BALF and serum through JNK1 signaling pathways and that JNK1 signaling pathways are closely related to MV-induced nitrite levels in the serum.

## Discussion

*A.b.* is commonly isolated from the hospital environment and hospitalized patients. Multiple factors tend to increase the risk of *Acinetobacter* infection, including previous antibiotic exposure, ICU admission, and use of catheter, MV, or hemodialysis. Imipenem therapy was the “gold standard” for pneumonia due to *A.b.* [25]. However, *A.b.* has rapidly developed resistance to most common types of antibiotics; therefore, the management of VAP caused by drug-resistant *A.b.* is still an ongoing challenge. The possible mechanism of lung injury caused by *A.b.* infection after MV was investigated by using mouse model receiving intranasal instillation of *A.b.* before MV. Our data demonstrated that intranasal instillation of 10^6^ CFU *A.b.* in C57BL/6 mice induced a significant increase in total cells and protein levels in the BALF and neutrophil infiltration in the lungs. MV after *A.b.* instillation increases neutrophil infiltration, expression of IL-6 and VCAM mRNA in the lungs, and total cells, IL-6 levels, and nitrite levels in the BALF. These results suggest that *A.b.* instillation alone can induce neutrophil infiltration in the lungs through the increase in inflammatory mediators and total cells in the BALF. MV further enhances *A.b.*–induced neutrophil infiltration through the increase of IL-6 and nitrite levels in the BALF and VCAM mRNA expression in the lungs. These results indicate that protein concentrations in the BALF are easily stimulated by *A.b.* instillation and that MV could stimulate VCAM mRNA expression in the lungs and levels of IL-6 and nitrite in the BALF through mechanism other than bacterial instillation.

In this study, WT mice with *A.b.* instillation (10^6^ CFU) and ventilation showed a considerable increase in lung MPO activity after ventilation but not with *P.a.* instillation. A significant increase was found in the number of cells in the BALF from mice with *A.b.* instillation but not with *P.a.* instillation group. After ventilation, the number of cells further significantly increased in mice with *A.b.* instillation but not with *P.a.* instillation group. AMs in C56B/6 mice showed a greater killing activity for *A.b.* than for *P.a.*. Altogether, these results suggest that *A.b.* alone can promote more AMs or neutrophils migration than *P.a.*. The total protein concentrations in the BALF of WT mice receiving *A.b.* instillation were significantly increased compared with saline instillation. Interestingly, *P.a.* instillation alone had no effect on the protein concentrations in the BALF. These results indicate that intranasal *A.b.* instillation can induce a more extensive inflammatory response than *P.a.* instillation as demonstrated by the protein concentrations in the BALF. These results further suggest that *A.b.* is better than *P.a.* in inducing inflammation in the BALF.

The production of several cytokines and chemokines was investigated by different assay methods. Although the increase in TNF-α, IL-1β or IL-6 production was not obvious as assayed by ELISA, the real-time polymerase chain reaction assay showed that *A.b.* instillation alone has no significant effect on the expression of TNF-α. The observation of Qiu et al*.* demonstrated that TNF-α was significantly higher in the lungs of C57BL/6 mice soon (4 h) after infection and depleted quickly after 24 h [6]. Moreover, the expression of TNF-α in the BALF or lung tissue had no significant difference with or without supplemented ventilation, indicating that the TNF-α is not a key player in VAP caused by *A.b.* Production of IL-6 is induced by mechanical stress [26]. Our data further demonstrated that the protein levels and mRNA expression of IL-6 was significantly increased in the lungs of mice receiving *A.b.* instillation only after MV, suggesting that IL-6 production is mainly induced by MV but not by bacterial instillation. Previous studies have shown that intranasal administration of MIP-2 induced recruitment of neutrophils into the lungs, which significantly enhanced the resistance of A/J mice to intranasal *A.b.* infection and tissue bacterial burden [6]. These results are consistent with our data that VCAM mRNA expression in the lungs of mice receiving *A.b.* instillation was increased after MV.

Qiu et al. [27] summarized the interaction between *A.b.* and found that the host innate immune system was likely to govern the extent of bacterial proliferation and local host inflammatory response following pulmonary bacterial infection. Although macrophages have a relatively minor role in the overall host defense against *A.b.*, they play an important role in the initial stage of host defense against *A.b.*-induced respiratory infection partially through an NO-dependent mechanism. In this study, *A.b.* instillation after MV induced a significant increase in nitrite levels in the BALF of mice. These observations suggest that induced inducible nitric oxide synthase (iNOS) expression may be involved in the tissue damage caused by *A.b.* after ventilation, a situation similar to the role of NO production in carbon-tetrachloride-induced acute liver injury [28].

In a previous study, *JNK1*^*−/−*^* mice* receiving *P.a.* instillation and ventilation showed decreased lung injury compared with WT mice, suggesting that JNK may be an important regulator in the expression of IL-6 in VAP caused by *P.a.* [29]. Therefore, JNK1^−/−^ mice were used to investigate the role of JNK activation in *A.b.* intranasal infection and ventilation in this study. Results of Li et al*. and* Limtrakul et al*.* [30, 31] indicated that the possible mechanism of lung damage caused by *A.b.* and ventilation was that *A.b.* instillation would increase the TNF-α production by AMs and activate the NF-κB pathway, which leads to an increased translation of IL-1β. The resultant TNF-α subsequently induces the inflammatory responses through the JNK signaling pathway and finally enhanced the translation of iNOS and IL-6 by activated AP-1. Our data demonstrated that the total cells in the BALF, nitrite levels in the BALF and nitrite levels in the serum were all lower in JNK1^−/−^ mice receiving *A.b.* instillation with or without ventilation than in WT mice. These results indicate a lower inflammation level in the lungs of JNK1^−/−^ mice.

Qiu et al*.* discovered that many AMs contained high numbers of bacteria within the cytoplasm, but only a few bacteria were visible in the cytoplasm of few neutrophils at 4 h post infection. The results indicated that *A.b.* induced moderate activation and recruitment of AMs into the lungs and AMs have the ability to clear bacteria immediately after infection [7]. Furthermore, a recent study indicated that TNF-α production was closely related to the killing ability (TNF-α-induced killing) [32]. In this study, the result of ex vivo stimulation of AMs isolated from WT mice showed a high level of TNF-α production when stimulated by live *P.a.*, but the TNF-α level was slightly lower when stimulated with live *A.b*. The ex vivo bacteria-killing activity of AMs also showed similar results with TNF-α production when stimulated with *P.a.* or *A.b*. The killing ability of AMs against *A.b.* was lower than against *P.a.*, and the ability was decreased regardless if it was against *P.a.* or *A.b.* after ventilation. Similarly, the ability of AMs to kill *A.b.* was lower than to kill *A.b.* after ventilation. The report of in vitro phagocytosis of *A.b.* by Qiu et al*.* showed that only 3% of the bacteria were detected at 4 h after incubation of *A.b.* with murine macrophage cell line J774A.1 [7]. Compared with the results described above, the bacterial count analysis showed that no colony was detected in the lungs of mice at 48 h after instillation with either *P.a.* or *A.b.*, indicating that most of the instilled bacteria were almost cleared in this model. Altogether, our data suggest that the killing activity of AMs against *A.b.* is lower than against *P.a.* MV treatment decreases AMs’ ability to kill *A.b.* and *P.a.*

Although macrophages have a relatively minor role in the overall host defense against *A.b.*, they play an important role in the initial stage of host defense against respiratory *A.b.* infection partially through an NO-dependent mechanism. In this study, there was a significant increase in the nitrite level in BALF of mice with *A.b.*instillation after ventilation. Inhibition of iNOS activity by SMT was demonstrated by a decrease in the nitrite level in BALF of mice receiving SMT injection at 1 or 2 h before ventilation. SMT significantly decreased the total number of cells in BALF. These observations suggest that induced iNOS expression may be involved in the tissue damage caused by *A.b.* after ventilation, a situation similar to the role of NO production in carbon-tetrachloride induced acute liver injury [28].

Our study had several limitations. First, we used a short-term and high stretch mechanical ventilation model (tidal volume, Vt = 30 ml/kg). Future studies using long-term ventilation model may have more clinical relevance. Also, we did not measure lung physiological parameters in our model such as hypoxemia, lung compliance, and airway resistance. We estimated lung infiltration of neutrophils based on MPO activity assay. The flow cytometry counting assay using Ly6G may be used to quantify mouse absolute number of neutrophils after instillation of *A.b.* with or without mechanical ventilation in the future.

In conclusion, *A.b.* induced a significant increase in the total cells and protein levels in the BALF and neutrophil infiltration in the lungs. MV after *A.b.* instillation increases neutrophil infiltration, IL-6 and VCAM mRNA expression in the lungs and total cells, IL-6 levels, and nitrite levels in the BALF. MV enhances *A.b.*-induced lung injury through the decrease in the killing activity of AMs and increase in VCAM expression and IL-6 levels in the BALF. Lower inflammation level and lung damage in JNK1^−/−^ mice indicates that *A.b.* VAP causes lung injury through JNK signaling pathway in the lungs.

## Supplementary Information

**Additional file 1.** Supporting information.

## Data Availability

C57BL/6 mice: National Laboratory Breeding and Research Center (NLBRC, Taipei, Taiwan)**.** JNK1^−/−^ mice: University of California, San Diego, CA, USA. Avertin: Sigma-Aldrich mechanical ventilator SAR-830/P: CWE Inc., Ardmore, PA, USA protein extraction buffer: Sigma-Aldrich proteinase inhibitor cocktail: Roche Life Science enhanced chemiluminescence detection reagent: Millipore.Biotinylated anti-mouse, anti-rabbit or anti-goat IgG: GenScript USA Inc. Mouse ELISA kit: eBioscience. Primary antibody for western blotting: R&D Systems.
