# Supplementary material for: Mechanical ventilation enhances Acinetobacter baumannii-induced lung injury through JNK pathways
Source: Respir Res. 2021 May 22;22:159. doi: 10.1186/s12931-021-01739-3 (PMC8140754; doi:10.1186/s12931-021-01739-3)
Supplement: Supplementary file 1 — Additional file 1. Supporting information. [file 12931_2021_1739_MOESM1_ESM.pdf]

Supporting information

**Primer sequences for qPCR**		
**Gene**	**Forward (5’ -3’)**	**Reverse (5’ -3’)**
Vcam	TTGGGAGCCTCAACGG TACT	GCAATCGTTTTGTATTCA GGGGA
Cxcr2	GGCGGGGTAAGACAAGAA TC	GGCAAGGTCAGGGCAAAGAA
Mip2	CCACTCTCAAGGGCGGTCAAA	TACGATCCAGGCTTCCCGGGT
Icam	GAGCTCAGCACTAGCACTTTGC	GTAGCGTGGGCTTGGCAC
Il1β	TGGTGTGTGACGTTCCCATT	CAGCACGAGGCTTTTTTGTTG
Il6	AAGCCAGAGTCCTTCAGAGAGA	ACTCCTTCTGTGACTCCAGCTT
Gapdh	AACTTTGGCATTGTGGA AGG	CACATTGGGGGTAGGAA CAC
Inos	CAGCTGGGCTGTACAAACCTT	CATTGGAAGTGAAGGGTTTCG
Tnfa	GAAAGGGGATTATGGCTCAGG	TCACTGTCCCAGCATCTTGTG
